# Comparison of the Allelic Alterations between InDel and STR Markers in Tumoral Tissues Used for Forensic Purposes

**DOI:** 10.3390/medicina57030226

**Published:** 2021-03-02

**Authors:** Pamela Tozzo, Arianna Delicati, Anna Chiara Frigo, Luciana Caenazzo

**Affiliations:** 1 Department of Molecular Medicine, Laboratory of Forensic Genetics, University of Padova, 35121 Padova, Italy; arianna.delicati@studenti.univr.it (A.D.); luciana.caenazzo@unipd.it (L.C.); 2 Department of Cardiac, Thoracic, Vascular Sciences and Public Health, University of Padova, 35121 Padova, Italy; annachiara.frigo@unipd.it

**Keywords:** human DNA identification, InDel markers, microsatellite instability, loss of heterozygosity, tumor samples, STR typing

## Abstract

*Background and objectives*: Over the last two decades, human DNA identification and kinship tests have been conducted mainly through the analysis of short tandem repeats (STRs). However, other types of markers, such as insertion/deletion polymorphisms (InDels), may be required when DNA is highly degraded. In forensic genetics, tumor samples may sometimes be used in some cases of human DNA identification and in paternity tests. Nevertheless, tumor genomic instability related to forensic DNA markers should be considered in forensic analyses since it can compromise genotype attribution. Therefore, it is useful to know what impact tumor transformation may have on the forensic interpretation of the results obtained from the analysis of these polymorphisms. *Materials and Methods*: The aim of this study was to investigate the genomic instability of InDels and STRs through the analysis of 55 markers in healthy tissue and tumor samples (hepatic, gastric, breast, and colorectal cancer) in 66 patients. The evaluation of genomic instability was performed comparing InDel and STR genotypes of tumor samples with those of their healthy counterparts. *Results*: With regard to STRs, colorectal cancer was found to be the tumor type affected by the highest number of mutations, whereas in the case of InDels the amount of genetic mutations turned out to be independent of the tumor type. However, the phenomena of genomic instability, such as loss of heterozygosity (LOH) and microsatellite instability (MSI), seem to affect InDels more than STRs hampering genotype attribution. *Conclusion*: We suggest that the use of STRs rather than InDels could be more suitable in forensic genotyping analyses given that InDels seem to be more affected than STRs by mutation events capable of compromising genotype attribution.

## 1. Introduction

STR typing is the most commonly-used approach in human DNA identification and kinship analysis due to its capacity of differentiation, reliability, and sensitivity. However, in the case of degraded DNA, the large size of polymerase chain reaction (PCR) amplicons may limit the analysis. The importance of analyzing other polymorphisms such as single nucleotide polymorphisms (SNPs) and insertion deletion polymorphisms (InDels) in forensic genetics has recently increased due to the small size of their amplicons that makes them a useful complementary tool of STR typing. InDels are the insertion or deletion of one or more nucleotides occurring in genome sequencing, thus resulting in DNA length variation. Therefore, InDel typing can be applied in forensic casework for ancestry inference, kinship analysis, disaster victims identification, reconstruction of the genetic structure of different human populations, analysis of degraded DNA, mixture deconvolution, and for the prediction of phenotypic traits commonly termed “External Visible Characteristics”, such as skin color, hair and eye color, height, weight, and facial morphology [1,2,3,4,5,6]. In particular, InDels may be very useful in forensic science since they combine the advantages of both STRs and SNPs such as short amplicon size, low mutation rate, high diffusion along the genome, and simple analysis due to the use of the same process adopted for STR typing (PCR followed by capillary electrophoresis (CE)) [7]. In 2009, a multiplex InDel analysis was described by Pereira et al. [8], combining 38 highly polymorphic loci in a single PCR. This multiplex combines all the advantages mentioned above regarding InDels. However, the population data for these new markers are still limited [9].

The biological samples that are used for identification purposes can come from tumor portions stored in biobanks when these are the last sources of biological material available. Generally, tumoral tissue specimens are not commonly used for forensic purposes since their mutations can interfere with the forensic genotype analysis. However, tumoral tissues are used when they represent the most advantageous of the options available. For instance, in paternity tests where the putative father is deceased, or in personal identification of unidentified bodies in mass casualty events [10].

With regards the tumor, carcinogenesis is a multistep process in which cells accumulate genetic alterations as they progress to a more malignant phenotype [11]. Specifically, genetic mutations in tumors were generally reported at the level of oncogenes and tumor suppressor genes [12], although, the entire genome can undergo mutations during the tumor transformation of a cell. In particular, these mutations can also affect the loci of those STR markers commonly used in forensic science, which are STRs usually associated with non-codifying regions of the genome. Moreover, despite their low mutation rate, sometimes, as occurs with STRs, InDels also undergo genetic changes in several diseases, such as tumors [10]. Overall, this genetic diversity of tumors must be considered during forensic studies since it could hamper the interpretation of the genotyping results.

In general, genomic instability in tumors can result in chromosomal instability (CIN) or microsatellite instability (MSI or MIN), depending on the type of genetic alteration [13]. MSI is characterized by an alteration in the length of the STRs resulting from the genetic or epigenetic inactivation of genes responsible for the maintenance of DNA integrity. MSI determines the appearance of an extra allele (entailing a shift from a homozygote genotype to a heterozygote genotype or from a bi-allelic genotype to a tri-allelic one) or of a new allele [10,13,14]. MSI in tumor samples can be divided into two different groups: Low-frequency MSI (MSI-L), when <33% of the analyzed loci display instability, and high-frequency MSI (MSI-H), when the instability appears in >33% of the analyzed loci [10,13,14].

The main type of genomic instability is chromosomal instability and is manifested as a loss of heterozygosity (LOH) which can sometimes be only partial (pLOH). It is possible to consider an allelic loss (LOH only if that ratio is lower than 0.5 or higher than 2.0). In some cases, it is more appropriate to speak of partial loss of heterozygosity (pLOH) when the peak height of one allele is >50% decreased in the tumor. In any case, forensic evaluations are complicated, with the possibility of a wrong genotyping, only when the tumors show MSI or complete loss of heterozygosity (cLOH) (12). In the case of STRs, some authors have already studied LOH and MSI, rationalizing the use of tumor samples for forensic purposes and selecting the most suitable polymorphisms and types of tumor to be used for the analysis [10,15,16,17,18].

The objective of this study is to analyze genomic instability in tumor samples considering InDel profiles compared to STR ones. In particular, the analysis aims to understand whether there are tumors, among those analyzed, which are subjected to greater variation in STRs and InDels; whether there are specific STRs or InDels which are more affected by mutations; and whether there is a significant difference in genomic instability between STRs and InDels. To our knowledge, this is the first work performed investigating genomic instability for forensic purposes based not only on traditional STRs but also on InDels. In order to do this, we have used the AmpFℓSTR^®^ NGM SelectTM PCR Amplification KitTM and InDelPlex INDEL Polymorphism Detection Kit for the analysis of STRs and InDels, respectively. As highlighted by Budimlija et al., it is important to evaluate the stability and reliability of alleles using genetically-altered biological material, especially before their application to archival tissues of pathological origin [19].

## 2. Materials and Methods

The study was performed on 132 tissue samples obtained from 66 individuals divided as follows:

- 38 samples from 19 individuals (three females and 16 males) with hepatic cancer: 19 frozen healthy tissues and 19 frozen tumors;
- 18 samples from nine individuals (three females and six males) with gastric cancer: Nine frozen healthy tissues and nine frozen tumors;
- 22 samples from 11 women with breast cancer: 11 frozen healthy tissues and 11 frozen tumors;
- 54 samples from 27 individuals (five females and 22 males) with colorectal cancer: 27 frozen healthy tissues and 27 frozen tumors.

The samples of colorectal, breast, and gastric cancer and the healthy tissue samples came from the biobank of the Department of Oncological and Gastroenterological Surgical Sciences of the University of Padua, while liver tissue samples (tumoral and healthy) came from the Liver Biobank of the Department of Medicine of the University of Padua. All the samples were delivered anonymously and encoded with the number attributed to the sample by the biobank. Both healthy and tumor frozen samples were stored at −80 °C.

Each healthy tissue was collected at the same surgical time as the tumor.

### 2.1. Sample Preparation

Each tissue sample consisted of about 25 mg of biological material which was entirely used for this study. In particular, the tissue was fragmented into small pieces by means of a mechanical pestle into a 1.5 mL microcentrifuge tube and was incubated according to the indications provided by the QIAamp^®^ DNA Micro Kit (Qiagen, GmbH, Hilden, Germany).

### 2.2. DNA Extraction

The genomic DNA was extracted from tissue samples following the manufacture protocol of the QIAamp^®^ DNA Micro Kit (Qiagen, GmbH, Hilden, Germany) with modification of the incubation period prolonged overnight.

An amount of 80 µL of Buffer AE was added and the eluate containing the purified DNA was obtained after 5 min of incubation at room temperature and 1 min of centrifugation at full speed.

### 2.3. DNA Amplification

After extraction, human DNA was quantified using Thermo Scientific™ NanoDrop™ One Microvolume UV-Vis Spectrophotometers. For STR genotyping purposes, amplification was carried out in GeneAmp PCR System 9700, 96-well gold-plated (Applied Biosystems, Foster City, CA, USA) using the AmpFℓSTR^®^ NGM SelectTM PCR Amplification KitTM and following the manufacturer’s procedure. This kit allows for the simultaneous amplification of 16 STR polymorphisms (D10S1248, vWA, D16S539, D2S1338, D8S1179, D21S11, D18S51, D22S1045, D19S433, TH01, FGA, D2S441, D3S1358, D1S1656, D12S391, SE33) and Amelogenin locus, for sex determination.

For the amplification, the manual provided by the kit company was followed with a minor modification related to the amount of the reagents. Firstly, the “Pre-Taq” program of the thermocycler was activated. The number of required tubes were positioned into the rack and they were opportunely signed with the correspondent sample code. The PCR Master Mix was prepared with the AmpFℓSTRTM NGM SElectTM Master Mix and AmpFℓSTRTM NGM SElectTM Primer Set. The total amount of Master Mix to prepare is given by the sum of the quantity of each single reagent necessary for the various samples. The amount of reagents for each sample is the following: 5 μL AmpFℓSTRTM NGM SElectTM Master Mix and 2.5 μL AmpFℓSTRTM NGM SElectTM Primer Set.

The tube containing the Master Mix was vortexed for 3 s and briefly centrifuged. The PCR Master Mix was equally aliquoted into each tube. According to the DNA quantification results, a total volume of 5 µL, composed by the sample DNA and water, was added in the respective tubes to obtain a final amount of the DNA template between 0.5 and 2 ng (the best result can be obtained with 1 ng of DNA template). A drop of mineral oil was added to each tube, the tubes were centrifuged for a few seconds and loaded into the thermocycler. The amplification reaction was run in the following conditions: 95 °C for 11 min; (94 °C–1 min/59 °C–2 min/72 °C–1 min) for 28 cycles; 60 °C for 60 min; 4 °C until the end of the reaction.

For the purpose of InDel genotyping, amplification was carried out in the GeneAmp PCR System 9700, 96-Well Gold-Plated (Applied Biosystems, Foster City, CA, USA) using the InDelPlex INDEL Polymorphism Detection Kit (Genomica, S.A.U., Grupo Zeltia, Madrid, Spain) following the manufacturer’s procedure. This kit allows for the simultaneous amplification of 38 InDel polymorphisms (rs3047269, rs2307579, rs16624, rs2308242, rs2308026, rs2307526, rs1160956, rs1610871, rs2307710, rs2307839, rs2308137, rs2307978, rs35769550, rs5895447, rs16402, rs2067294, rs2307580, rs140809, rs1160886, rs10688868, rs34811743, rs33972805, rs1610919, rs2067238, rs2308171, rs2308189, rs2308020, rs2067208, rs3051300, rs3080855, rs34511541, rs36040336, rs2307689, rs33917182, rs34541393, rs35605984, rs10629077, and rs2307700; mapping data according to dbSNP build 150).

The preparation of the sample for the amplification requires: 5 µL of Qiagen Multiplex PCR master mix (2×), 1 µL of Primer mix (10×), and 3 µL of RNAse Free water for each sample. The tube containing the Master Mix was vortexed for 3 s and briefly centrifuged. The PCR Master Mix was equally aliquoted into each tube. The total reaction volume of 10 µL was reached by adding 1 µL of template DNA, which previously led to a concentration of 0.5 ng/µL. A drop of mineral oil was added into each tube and the tubes were centrifuged for a few seconds and loaded into the thermocycler. The amplification reaction was run in the following conditions: 95 °C for 15 min; (94 °C–30 s/60 °C–90 s/72 °C–45 s) for 10 cycles; (94 °C–30 s/58 °C–90 s/72 °C–45 s) for 20 cycles; 72 °C for 60 min; 10 °C until the end of the reaction.

### 2.4. STR Genotyping

The analysis of the amplified DNA samples was conducted using the 3130 Genetic Analyzer (Applied Biosystems, Foster City, CA, USA). All the samples were prepared in the same way. Two tubes (one for STR and one for InDel analysis) were filled with:

- Hi-DiTM formamide: 15 µL × (number of the sample + 1);
- Specific internal standard (GeneScanTM–600 LIZ^®^ Size Standard for STRs and GeneScanTM—500 LIZ^®^ Size Standard for InDels): 0.5 µL × (number of the sample + 1).

The tubes were vortexed and centrifuged briefly. A total of 15 µL of the two solutions were aliquoted into the appropriate wells of MicroAmp^®^ Optical 96-well reaction plates. Then, for STR purposes, 1 µL of the sample or allelic ladder was loaded into its respective well and the plate was loaded into the sequencer. Whereas, for InDel purposes, only the addition of 1 µL of the sample in each well was conducted before loading the plate into the sequencer. Both final genotyping results were produced using GeneMapper ID Software version 3.2 (Applied Biosystems, Foster City, CA, USA).

Eventually, all the data were elaborated with the software GraphPad Prism Software version 8.4.3 (GraphPad Software Inc., La Jolla, CA, USA) and the statistical evaluation was tested with a Chi-Square test. The level of significance was set at the 5% level.

## 3. Results

This study evaluated the genomic instability in InDels and STRs in tumor samples compared with their healthy tissue counterparts, in the context of human identification for forensic purposes, in order to understand whether there are any differences between the mutational events in these two groups of markers. Our objective was to evaluate whether the typing differences in tumor tissues compared to healthy counterparts were more impactful for identification purposes for STR or InDel polymorphisms.

To clarify our results, a premise on how we chose the types of alterations to be evaluated is appropriate. The alterations which were considered are LOH, pLOH, and MSI.

Technically, MSI should be evaluated only for STRs, which are these microsatellites, while for InDels the appearance of a new allele or an additional allele can be observed. For InDel polymorphisms, we have considered, for terminological uniformity, as MSI the appearance of a new allele or the substitution of an allele: In particular, for InDel markers, microsatellite instability was considered in cases where the homozygous genotype in healthy tissue (1,1 or 2,2) changed in the tumor tissue resulting in a heterozygosity or a different homozygosity (change from 1,1 to 2,2 or vice versa). Microsatellite instability was identified by comparing the genotype at each STR and InDel locus in the frozen tumor sample with the genotype obtained from the respective healthy frozen tissue. Allelic deletion in the frozen tumor tissue as compared with its respective heterozygotic frozen healthy tissue, was classified as “LOH” both for STRs and InDels. Moreover, LOH was attributed to a peak intensity ratio (peak ratio in the tumor tissue/peak ratio in the healthy tissue) of <0.5. We considered complete loss of heterozygosity (cLOH) only if that ratio turns out to be lower than 0.5 or higher than 2.0, while a partial loss of heterozygosity (pLOH) was identified when the peak height of one allele was >50% decreased in tumor.

All polymorphisms of all samples were amplified in duplicate experiments and the results were concordant for all the samples. Samples without alterations (MSI, LOH) showed an identical profile between the healthy and tumor tissue and the duplicates of samples where pLOH was established showed no difference in peak height ratios.

### 3.1. STR Results

**Figure 1:**
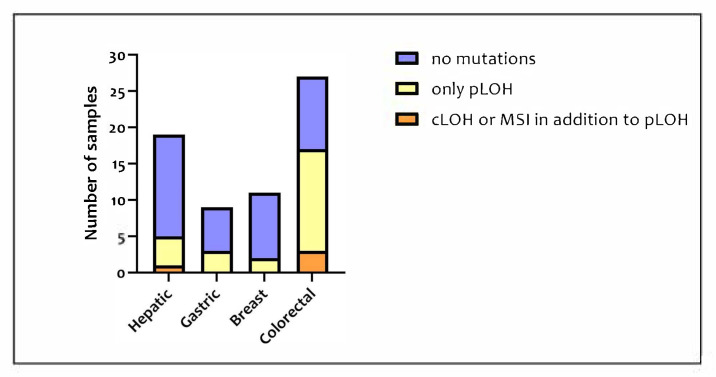
Number of samples in short tandem repeats (STR) typing divided based on both tissue and mutation types.

An Amelogenin marker and the 16 STRs of the AmpFℓSTR^®^ NGM SelectTM PCR Amplification KitTM were studied for each of the 132 samples available, for a total of 2244 loci analyzed. Among the 66 tumor samples, mutational events were observed in 27 samples (40.9%). In Figure 1, the proportion of tumor samples presenting no mutations, only pLOH, and pLOH with LOH and MSI is shown for each type of tissue included in this study.

The number of altered polymorphisms for each sample varied from 1 to 6, considering all the loci and the type of mutations. The samples with the highest number of alterations were a gastric tumor and a colorectal one. LOH was the most frequent alteration but no locus showed the total loss of both alleles. The samples most affected by LOH were the colorectal tumors, which were mutated in 17 cases out of 27. Whereas, in the other tumor types, LOH occurred in five cases out of 19 in hepatic tissue, three cases out of nine in gastric tissue, and two cases out of 11 in breast tissue.

On the basis of the analysis of the mutated and non-mutated loci, the colorectal tumor appeared to be affected by the highest number of mutations hampering or preventing the correct attribution of the STR genotype. A first comparison between the mutated and non-mutated loci of hepatic, gastric, and breast tumors did not show a significant difference between the different types of tumors (*p* = 0.4093). However, the result became statistically significant (*p* = 0.0002) when the data of the colorectal tumor were added to the analysis.

The STR data are summarized in Table 1, differentiated on the basis of the different loci analyzed and of the tissue type.

**Figure 2:**
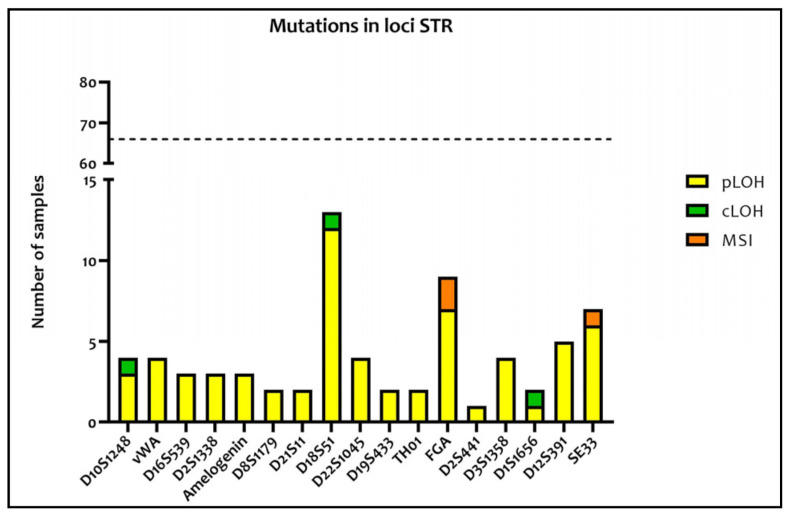
Graphical representation of the mutation distribution among the different STR loci. The polymorphisms mutated in the majority of samples were D18S51 (12 partial loss of heterozygosity (pLOH) and one complete loss of heterozygosity (cLOH)), FGA (seven pLOH and two microsatellite instability (MSI)), and S33 (six pLOH and one MSI). The dotted line indicates the total number of samples which were analyzed, so the difference between the dotted line and the height of each column represents the number of samples not mutated for that particular polymorphism.

The most altered polymorphisms were: D18S51 (12 pLOH and one LOH), FGA (seven pLOH and two MSI), and SE33 (six pLOH and one MSI). The Amelogenin locus showed only three pLOH individually distributed into three different colorectal tumoral samples: Even if Amelogenin is not a STR, it is important to consider these alterations as they could lead to an incorrect attribution of sex in the tumor tissue (Figure 2).

### 3.2. InDel Results

**Figure 3:**
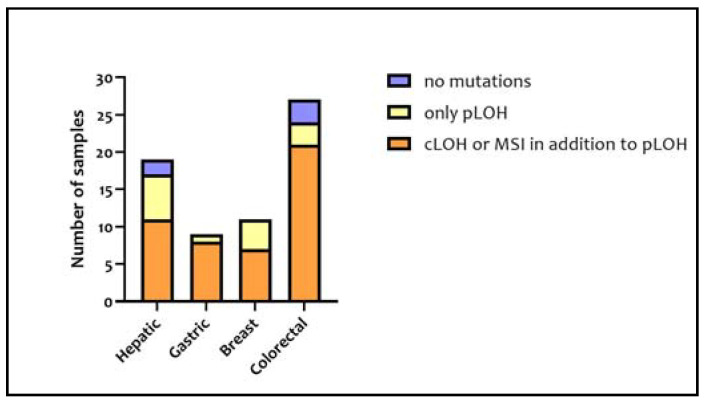
Number of samples in insertion/deletion polymorphisms (InDel) typing classified based on both tissue and mutation types.

The 38 InDel polymorphisms of the InDelPlex INDEL Polymorphism Detection Kit were analyzed in each sample for a total of 5016 loci analyzed. Specifically, 17 hepatic, nine gastric, 11 breast, and 24 colorectal samples, which overall represent 92.4% of the samples, reported mutations altering the tumor-sample genotype in comparison to their corresponding healthy tissue. Among the mutated samples, the ones with LOH or MSI in addition to pLOH numbered 47. In Figure 3, the proportion of tumor samples presenting no mutations, only pLOH, and pLOH with LOH and MSI is shown for each type of tissue included in this study.

Only five tumor samples (two hepatic and three colorectal) did not show any mutations. Among the remaining, the samples with the highest number of mutations were a breast tumor and a hepatic tumor.

**Figure 4:**
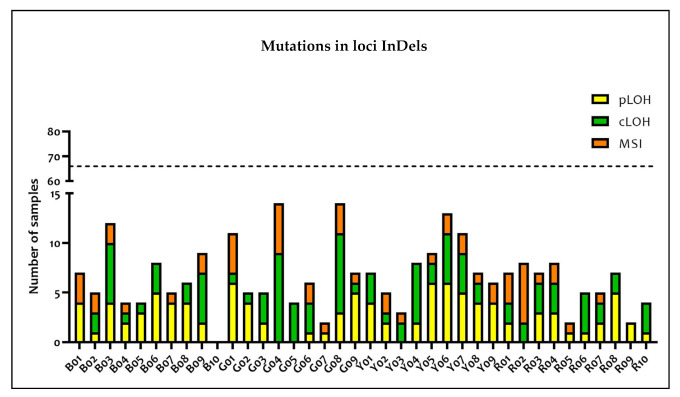
Graphical representation of the mutation distribution among the different InDel loci. The polymorphisms mutated in the majority of samples were G04 (nine cLOH and five MSI), G08 (three pLOH, eight cLOH, and three MSI), Y06 (six pLOH, five cLOH, and two MSI), and B03 (four pLOH, six cLOH, and two MSI). Only B10 did not show any mutation. The dotted line indicates the total number of samples which were analyzed, so the difference between the dotted line and the height of each column represents the number of samples not mutated for that particular polymorphism.

The hypothesis that the InDels mutations depended on the type of tissue was rejected. Indeed, the comparison between the different amounts of InDel mutations in the different types of tissues turned out to be statistically insignificant (*p* = 0.9533). The mutations associated with each analyzed locus are represented in Figure 4.

In particular, the most altered loci were G04 (nine cLOH and five MSI), G08 (three pLOH, eight cLOH, and three MSI), Y06 (six pLOH, five cLOH, and two MSI), and B03 (four pLOH, six cLOH, and two MSI). LOH and MSI occurred 200 times (~8%) and 52 times (~2%), respectively. Moreover, at least one LOH was detected in each polymorphism except for B10 which did not show any alteration both in terms of LOH and MSI. Conversely, at least one MSI was observed in approximately 66% of the polymorphisms.

The InDels data are summarized in Table 2, differentiated on the basis of the different polymorphisms and of the tissue type.

**Figure 5:**
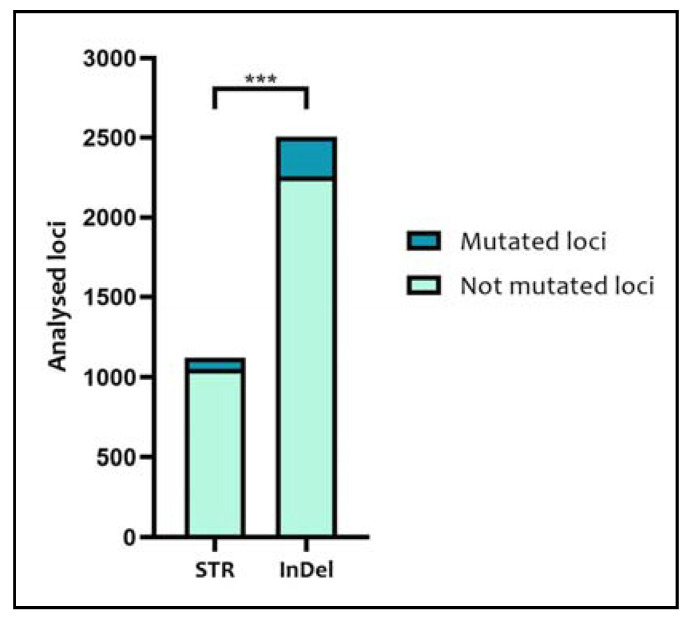
Comparison among the total number of mutated and non-mutated loci of STRs and InDels. The comparison was performed with the Chi-Square test; “***” = *p* < 0.001.

Considering the 66 tumor samples, 1122 STR loci and 2508 InDel loci were analyzed, showing, genotype alterations in 70 and 252 loci, respectively (Figure 5). Comparing these data, the difference in the number of mutated loci with respect to the total number of loci analyzed between STRs and InDels turned out to be statistically significant (*p* = 0.0002).

## 4. Discussion

The importance of InDels in forensic genetics has gradually increased in recent years, in particular, since their amplicon size is smaller than that of STRs and thus they may be suitable for highly degraded DNA samples. Furthermore, their low mutation rate is important in inheritance cases and their abundance and wide distribution along the genome make them a potential tool for analysis in forensic caseworks. However, the disadvantages of these polymorphisms are that they are bi-allelic and consequently less polymorphic and less informative—thus requiring a large panel of InDels for genotyping as compared to STRs. For this purpose, different kits of multiplex InDels were developed. In this study, we evaluated the efficacy of the InDelPlex INDEL Polymorphism Detection Kit in detecting mutation in tumor tissues.

On the basis of the results of the amplification of 16 autosomal STR and Amelogenin locus in 132 samples, 40.9% of tumor samples reported a mutated genotype. In particular, with regards to the gastrointestinal samples (27 colorectal and nine gastric), mutations were found in 55.6% of the cases in accordance with previous findings in the literature. Vauhkonen et al. analyzed 41 gastrointestinal tumoral samples showing mutations in 68% of the cases [16]; Pelotti et al. found mutations in 66% of 56 analyzed sporadic gastrointestinal carcinomas [17]; Li et al. determined that 64% of the colorectal tumoral samples showed mutations [18]; Ananian et al. analyzed both frozen and formalin-fixed tumors finding mutations in 54.6% of the frozen samples cases [10].

Regarding STRs, the gastrointestinal tumor samples showed a higher genomic instability than breast and hepatic tumor samples. Indeed, mutations were detected in two out of 11 breast tumor samples (18%) and in five out of 19 hepatic tumor samples (26%). With regards to breast tumors, the frequency of STR mutations appears to be relatively low, as borne out by previous studies [7,19]. To our knowledge, previous studies concerning STR genomic instability in hepatic tumors are lacking. Therefore, further studies are needed to confirm the reliability of our result.

The slight differences between our study and all the others are likely due to the different types of tumors analyzed and to the different amplification kits adopted, employing different sets of polymorphisms.

It should be noted that the samples in which the genotype attribution is prevented are those with polymorphisms affected by MSI or LOH since the former determines the appearance of an extra allele or of a new allele and the latter leads to the total loss of an allele (false homozygosis). Instead, pLOH results only in an imbalance peak height with one allele reduced by more than 50% [12]. Among the 66 tumor samples, only four samples (6%) were affected by MSI or LOH in at least one STR: Three colorectal tumors and one hepatic tumor. Based on these considerations, the samples can be divided into three groups: The tumor samples without mutations which guarantee a correct genotype attribution; the tumor samples with only pLOH which may allow a correct genotype attribution; and the tumor samples with MSI or cLOH in addition to pLOH which do not allow a correct genotype attribution.

LOH represents the most frequent mutation in STRs, including in our study. Specifically, 91.4% of mutations that we detected was pLOH, whose relevance, nevertheless, is not so great in the forensic field given that pLOHs do not prevent correct genotype attribution (12). The main discrepancy between our work and previous studies is associated with the frequency of MSI—given that we detected only three MSI events, which resulted in an STR loci mutation rate of 3% for FGA and 1.5% for SE33. Conversely, Vauhkonen et al. found several loci affected by MSI with a mutation rate of 15–17% [16] and Pelotti et al. [17] confirmed the high incidence of MSI with a mutation rate of 3.6–16.1% (except for TH01 which turned out to be stable).

In our study, D18S51, FGA, and SE33 turned out to be the most mutated STRs with, respectively, 13, 9, and 7 total mutation events. In this sense, our results seem to confirm the conclusion of different studies which showed that D18S51 and FGA represent two of the most frequently mutated loci [10,16,17,18,20]. All these studies were mainly focused on gastrointestinal tumors. Therefore, the fact that D18S51 and FGA were the most mutated STRs is probably valid only for these tumor types given that, in our case, 54% of the samples were made up of gastrointestinal tissue. The proximity of these STR loci to onco-suppressor genes, which are altered during colorectal tumor progression, could be one of the reasons for the higher level of their genetic instability. In particular, D18S51 (18q21.33), FGA (4q31.3), and SE33(6q14) are near some regions which frequently undergo deletions in gastrointestinal tumors such as 5q22.2 (APC gene), 17p13.1 (TP53 gene), 18q (DCC, SMAD2 and 4, cables) [21], 4q28.3 (protocadherin 10) [22], and different portions of the six chromosomes associated with genes mutated in colorectal tumors.

Finally, another parameter to evaluate is the degree of mutation of the Amelogenin gene. We detected only three pLOH in an equal number of male colorectal samples. No total loss of Y chromosome (LOH) was observed. This is important since a total loss of the Y chromosome can result in an incorrect sex attribution. However, problems related to the Amelogenin gene may not represent an issue when sex can be detected and confirmed with other forensic technologies.

In the InDel analysis, 61 out of 66 tumor samples (92.4%) were characterized by at least one mutation and only five samples (two hepatic and three colorectal) showed no mutations. However, only 47 samples (71.2%) were found to be affected by mutations which prevented genotype attribution. Analyzing InDel genotypes of tumor and healthy tissues, it should be noted that the majority of the mutations were LOHs (79.4% of all the mutations) of which 41.7% were only partial (pLOH). MSI events constituted only 20.6% of all the mutations. Despite the fact that some InDels turned out to be more mutated than others, the mutations seemed to be equally distributed across all the InDel loci with the sole exception of B10, which was not affected by any MSI or LOH event.

On the basis of a statistical comparison of the mutations that occurred in the 38 InDel loci of the 132 samples, no significant differences were noted between the different tissues, supporting the idea that InDels mutate in a manner which is independent of the tumor type.

Considering STR and InDel analysis, a total of 55 markers for each sample was analyzed and a substantial difference between mutated and non-mutated samples of the two groups of markers was detected. In STRs, only 70 out of 1122 loci turned out to be mutated as compared to InDels where mutations occurred in 252 out of 2508 loci, showing a great difference between the percentages of the mutated samples, 40.9% in STRs and 92.4% in InDels.

Moreover, the higher number of both MSI and LOH in InDels than in STRs is reflected by the higher number of samples that showed different profiles in healthy and tumoral tissues. Indeed, 62 out of 66 samples showed the same STR profile, whereas, only 19 showed the same InDel profile.

Our results appear to be the opposite of those identified by Zhao et al. in 2011 [23]. These authors considered the InDels in gastrointestinal tumor tissues to be more stable than STRs. The differences between our results and those reported by Zhao et al. can be attributed to the fact that different polymorphisms have been analyzed: In our sample, we also included non-gastrointestinal tumors and hepatic cancer and the reference population is different (Italian patients and Chinese patients). All these elements can justify the difference in the results in these two studies and, at the same time, can stimulate further research in this field in order to arrive at an unambiguous affirmation of the greater stability or instability of STRs compared to InDels.

In routine caseworks, if no other biological material is available to obtain genetic profiles of forensic interest, it has been proposed that the peritumoral samples be taken through histopathological analysis of the sample to be genotyped [17]. However, it should be considered that, even in this tissue, genetic alterations may be present not only at the level of the STRs, as already reported in the literature [10,15,16,17,18], but also at the level of the InDels. In fact, in the light of our considerations, the forensic geneticist will have to pay greater attention to the interpretation of the tumor samples results.

In order to obtain more reliable results and to be able to interpret them, it would thus be appropriate to perform histological analyses before forensic genotyping and set up PCR multiplexes analyzing different InDels in order to strengthen the robustness of genetic information.

According to the type of tumor, a careful selection of the markers to be analyzed should be done in order to identify the most stable loci and those which are more distant from the regions which generally mutate in that tumor. If this is not possible, a higher number of markers should be considered, placing a different importance on the markers based on their stability. Moreover, to ensure more reliable results, if the tumor is the only available source which can be used for forensic investigations, a careful histological exam should be performed on the samples to isolate possible areas of healthy tissue. This should guarantee a more similar result to the one obtained with the healthy tissues.

The results of this study are promising, even if some limits should be considered, such as the use of only frozen tissues, the limited number of tumor types and the absence of archival pathology samples as paraffin-embedded tissues, which are more frequent in real caseworks and that would be useful to evaluate also the effect of DNA degradation on genetic instability.

## 5. Conclusions

In conclusion, a tumor is sometimes the only available source of DNA in order to perform forensic genetics analysis, such as paternity and human DNA identification tests. Considering our results altogether, we suggest that the use of STRs rather than InDels could be more suitable in forensic genotyping analyses, given that InDels seem to be more affected than STRs by mutation events capable of compromising genotype attribution. The considerations made hitherto have demonstrated the importance of exercising great caution in genotyping and data interpretation when tumors are used in the forensic field.

## Figures and Tables

**Table 1:** Number of mutational events found in each STR locus differentiated based on the tumoral type.

Title	Hepatic Cancer			Gastric Cancer			Breast Cancer			Colorectal Cancer			Total Number of Mutational Events
loci	pLOH	cLOH	MSI	pLOH	cLOH	MSI	pLOH	cLOH	MSI	pLOH	cLOH	MSI	
D10S1248	2									1	1		3 pLOH–1 cLOH
vWA	1									3			4 pLOH
D16S539				1			1			1			3 pLOH
D2S1338							1			2			3 pLOH
Amelogenin										3			3 pLOH
D8S1179				2									2 pLOH
D21S11	1									1			2 pLOH
D18S51				1						11	1		12 pLOH–1 cLOH
D22S1045	1						1			2			4 pLOH
D19S433				1						1			2 pLOH
TH01										2			2 pLOH
FGA			1	1						6		1	7 pLOH–2 MSI
D2S441							1						1 pLOH
D3S1358	1						1			2			4 pLOH
D1S1656							1				1		1 pLOH–1 cLOH
D12S391	2									3			5 pLOH
SE33				2			1			3		1	6 pLOH–1 MSI
Total	8 pLOH-1 MSI			8 pLOH			7 pLOH			41 pLOH–3 cLOH–2MSI			64 pLOH–3 cLOH–3MSI

STR: short tandem repeats; MSI: miscrosatellite instability; pLOH: partial loss of heterozigosity; cLOH: complete loss of heterozigosity.

**Table 2:** Number of mutational events found in each InDel locus differentiated based on the tumoral type.

Title	Hepatic Cancer			Gastric Cancer			Breast Cancer			Colorectal Cancer			Total Number of Mutational Event
loci	pLOH	cLOH	MSI	pLOH	cLOH	MSI	pLOH	cLOH	MSI	pLOH	cLOH	MSI	
B01			1	1			1			2		2	4 pLOH–3 MSI
B02		1	1	1							1	1	1 pLOH–2 cLOH–2 MSI
B03	1	2		1	1	1	1	1		1	2	1	4 pLOH–6 cLOH–2 MSI
B04				1			1		1		1		2 pLOH–1 cLOH–1 MSI
B05							3				1		3 pLOH–1 cLOH
B06	2			1						2	3		5 pLOH–3 cLOH
B07	2		1				2						4 pLOH–1 MSI
B08	1	1					2			1	1		4 pLOH–2 cLOH
B09	1	1							1	1	4	1	2 pLOH–5 cLOH–2 MSI
B10													
G01	1		1	2	1	1	1			2		2	6 pLOH–1 cLOH–4 MSI
G02	1	1					1			2			4 pLOH–1 cLOH
G03	1	1					1				2		2 pLOH–3 cLOH
G04		2	2		4			2	1		1	2	9 cLOH–5 MSI
G05		2						1			1		4 cLOH
G06			1		2			1		1		1	1 pLOH–3 cLOH–2 MSI
G07			1							1			1 pLOH–1 MSI
G08		2	1		1		2		1	1	5	1	3 pLOH–8 cLOH–3 MSI
G09	3			2							1	1	5 pLOH–1 cLOH–1 MSI
Y01	1	2		1						2	1		4 pLOH–3 cLOH
Y02	1					1			1	1	1		2 pLOH–1 cLOH–2 MSI
Y03		1			1							1	2 cLOH–1 MSI
Y04	1	3					1				3		2 pLOH–6 cLOH
Y05	2			1	2	1	2			1			6 pLOH–2 cLOH–1 MSI
Y06	2			1				1	1	3	4	1	6 pLOH–5 cLOH–2 MSI
Y07	2	1	1		1			2	1	3			5 pLOH–4 cLOH–2 MSI
Y08	1						1			2	2	1	4 pLOH–2 cLOH–1 MSI
Y09	1		1							3		1	4 pLOH–2 MSI
R01		1	3					1		2			2 pLOH–2 cLOH–3 MSI
R02			2			1		1	1		1	2	2 cLOH–6 MSI
R03		2	1				2			1	1		3 pLOH–3 cLOH–1 MSI
R04	1	1		1					1	1	2	1	3 pLOH–3 cLOH–2 MSI
R05			1	1									1 pLOH–1 MSI
R06	1				1					1	2		2 pLOH–3 cLOH
R07				1		1				1	2		2 pLOH–2 cLOH–1 MSI
R08							3			2	2		5 pLOH–2 cLOH
R09	1						1						2 pLOH
R10	1	1						1			1		1 pLOH–3 cLOH
Total	28 pLOH–25 cLOH–18 MSI			15 pLOH–14 cLOH–6 MSI			25 pLOH–11 cLOH–9 MSI			37 pLOH–45 cLOH–19 MSI			105 pLOH–95 cLOH–52 MSI

## Data Availability

The data presented in this study are available on request from the corresponding author.
